# Anti-Adipogenic Effects of Salicortin from the Twigs of Weeping Willow (*Salix pseudolasiogyne*) in 3T3-L1 Cells

**DOI:** 10.3390/molecules27206954

**Published:** 2022-10-17

**Authors:** Hee Jung Kim, Da Eun Lee, Eon Chung Park, Moon-Jin Ra, Sang-Mi Jung, Jeong-Nam Yu, Sung Hee Um, Ki Hyun Kim

**Affiliations:** 1 Department of Molecular Cell Biology, Samsung Biomedical Research Institute, Sungkyunkwan University School of Medicine, Suwon 16419, Korea; 2 Department of Health Sciences and Technology, Samsung Advanced Institute for Health Sciences and Technology, Samsung Medical Center, Sungkyunkwan University, Seoul 06351, Korea; 3 School of Pharmacy, Sungkyunkwan University, Suwon 16419, Korea; 4 Hongcheon Institute of Medicinal Herb, Hongcheon-gun 25142, Korea; 5 Nakdonggang National Institute of Biological Resources, Sangju 37242, Korea; 6 Biomedical Institute Convergence, Sungkyunkwan University, Suwon 16419, Korea

**Keywords:** *Salix pseudolasiogyne*, salicortin, 3T3-L1 cells, adipocyte differentiation, lipid metabolism

## Abstract

*Salix pseudolasiogyne* (*Salicaceae*), the “weeping willow,” has been used in traditional Korean medicine to treat pain and fever due to its high concentrations of salicylic acid and salicin. The present study investigated bioactive compounds from *S. pseudolasiogyne* twigs to discover bioactive natural products. Phytochemical investigation of the ethanol (EtOH) extract of *S. pseudolasiogyne* twigs followed by liquid chromatography–mass spectrometry (LC/MS)-based analysis led to the isolation of two salicin derivatives, salicortinol and salicortin, the structures of which were determined by interpretation of their NMR spectra and data from the LC/MS analysis. To the best of our knowledge, this is the first report of salicortinol isolated from *S. pseudolasiogyne*. The isolated compounds were evaluated for their anti-adipogenic effects in 3T3-L1 cells. Both salicortinol and salicortin were found to significantly inhibit adipocyte differentiation in 3T3-L1 cells. In particular, salicortin exhibited a strong inhibitory effect on lipid accumulation. Furthermore, salicortin inhibited the expression of lipogenic and adipogenic transcription factors, including FASN, FABP4, C/EBPα, C/EBPβ, and PPARγ, without inducing cytotoxicity. These results suggest that salicortin could be a potential therapeutic compound for the prevention or treatment of metabolic disorders such as obesity.

## 1. Introduction

Obesity is associated with life-threatening diseases including type 2 diabetes, cardiovascular disease, certain types of cancer, and hepatic diseases [1,2]. Obesity is characterized by the accumulation of excessive body fat caused by an increase in the number and size of differentiated mature adipocytes [3]. An increase in the number of adipocytes is called adipogenesis; it is related to the expansion of adipose tissue through the proliferation and differentiation of adipocyte precursor cells [4]. Adipogenesis is a major process that determines the number of adipocytes, which are mainly formed during childhood and adolescence, and determines the lipid storage capacity of fat mass and adipose tissue in adults [5,6,7]. Adipocyte differentiation involves multiple processes including changes in hormone sensitivity and morphology; it is regulated by transcriptional factors and signaling pathways [8]. Thus, identifying regulatory compounds and their underlying mechanisms to control adipocyte differentiation or proliferation could be an essential strategy to prevent obesity.

*Salix pseudolasiogyne* H. Lev., commonly known as the weeping willow, is mostly distributed in Asian countries, including the Republic of Korea. This plant belongs to the family *Salicaceae* and the genus *Salix*; the willow trees among *Salix* spp. have been used as medicinal herbs for the treatment of pain, fever, and inflammation for thousands of years [9]. *S. pseudolasiogyne* has also been used in traditional Korean medicine for the treatment of pain and fever [10]. In general, the bark of willow trees contains anti-inflammatory natural products, such as salicylic acid, a well-known natural source of aspirin, and salicin, a precursor of salicylic acid that has been widely used because it has a less direct action on cyclooxygenase than salicylic acid itself [11,12]. According to recent phytochemical studies on *S. pseudolasiogyne*, salicin is the primary constituent of *S. pseudolasiogyne* twigs, and salicin derivatives, such as 2′-*O*-acetylsalicin, salicortin, 2′-*O*-acetylsalicortin, 3′-*O*-acetylsalicortin, and 6′-*O*-acetylsalicortin, were mainly identified in the MeOH extract of *S. pseudolasiogyne* [13,14]. Interestingly, salicin derivatives bearing a 1-hydroxy-6-oxo-2-cyclohexenecarboxylate moiety, including salicortin, 2′,6′-*O*-acetylsalicortin, 2′-*O*-acetylsalicortin, 3′-*O*-acetylsalicortin, and 6′-*O*-acetylsalicortin, were reported to inhibit lipopolysaccharide-induced nitric oxide production in BV2 microglial cells in vitro [13]. In addition, the MeOH extract of *S. pseudolasiogyne* was reported to exert a cognitive-enhancing effect on scopolamine-induced memory deficits in mice and to reduce glutathione reductase levels, and 2′-*O*-acetylsalicortin was identified as the responsible bioactive compound [13]. However, the bioactive phytochemicals from *S. pseudolasiogyne* have not yet been fully investigated.

As part of our continuing research, we have discovered bioactive natural products from diverse natural sources [15,16,17,18,19,20]. We investigated the potential bioactive compounds in an ethanol (EtOH) extract of *S. pseudolasiogyne* twigs to search for anti-adipogenic constituents in the present study. Phytochemical investigation of the EtOH extracts, followed by liquid chromatography–mass spectrometry (LC/MS)-based analysis, led to the isolation of two salicin derivatives, salicortinol (**1**) and salicortin (**2**). The structures of the isolated compounds were determined using data from NMR experiments and LC/MS analysis, and the compounds were evaluated for their anti-adipogenic effects in 3T3-L1 cells. Herein, we describe the separation and structural elucidation of compounds **1** and **2**, evaluate their anti-adipogenic biological activities, and their mechanism of action.

## 2. Results

### 2.1. Isolation and Identification of Compounds

**Figure 1:**
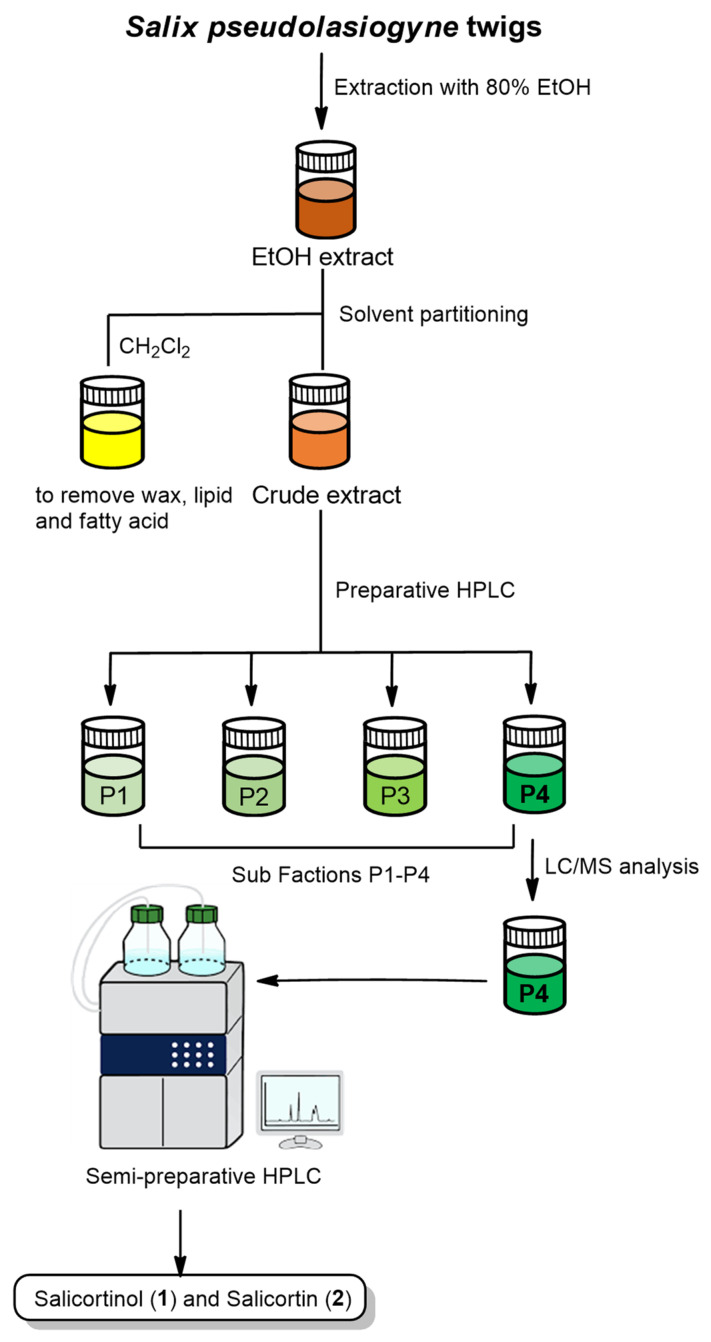
Separation scheme leading to salicortinol and salicortin identification.

**Figure 2:**
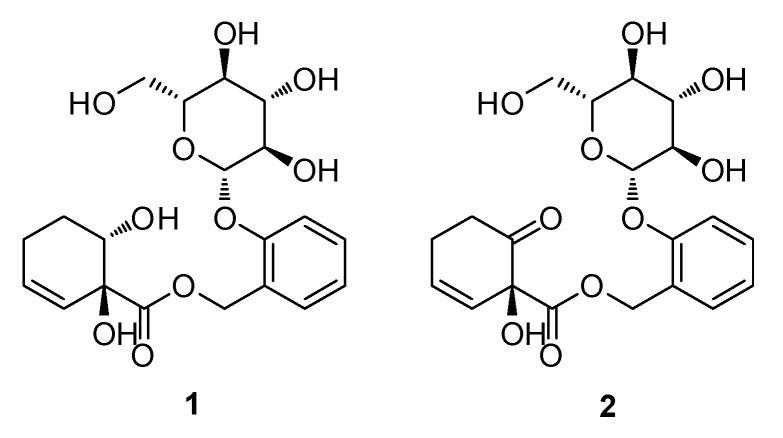
Chemical structure of salicortinol (**1**) and salicortin (**2**).

The EtOH extract of *S. pseudolasiogyne* twigs was subjected to solvent partitioning using CH_2_Cl_2_ to remove wax, lipids, and fatty acids prior to an LC/MS analysis of the fraction. The resultant extract was fractionated by preparative reversed-phase high-performance liquid chromatography (HPLC) to obtain four fractions (P1–P4) (Figure 1). Based on the results of the LC/MS analysis of the fractions, fraction P4 was further investigated by semi-preparative reversed-phase HPLC separation owing to the presence of the promising ion peaks expected for salicin derivatives. HPLC analysis led to the isolation and identification of two salicin derivatives (**1** and **2**) (Figure 2). Compounds **1** and **2** were identified as salicortinol [21] and salicortin [22], respectively, by comparing their NMR spectra with those previously reported in the literature and by analyzing the data from the LC/MS analysis (Figure S1 and S2). The absolute configurations of salicortinol (**1**) and salicortin (**2**) were confirmed by the specific rotation values of **1** [${\left[a\right]}_{D}^{25}$-33.2 (*c* 0.01, MeOH)] and **2** [${\left[a\right]}_{D}^{25}$-52.5 (*c* 0.01, MeOH)] comparable with those previously reported, ${\left[a\right]}_{D}^{25}$-72.26 (*c* 1.22, MeOH) for salicortinol [21] and ${\left[a\right]}_{D}^{22}$-123.9 (*c* 0.72, MeOH) for salicortin [22].

### 2.2. Effects of the Isolated Compounds on Lipid Accumulation

According to a recent study, the MeOH extract of *S. pseudolasiogyne* twigs reduced lipid accumulation in 3T3-L1 preadipocytes, and the anti-adipogenic constituents of such twigs were salicortin derivatives [14]. In particular, the mechanism of action of 2′,6′-*O*-acetylsalicortin, the most potent antiadipogenic compound, involved the downregulation of SREBP1c and C/EBPα dependent pathways [14]. Thus, it was necessary to determine whether salicortinol and salicortin, the compounds isolated and identified here, showed any antiadipogenic effect, and to fully understand such effect.

**Figure 3:**
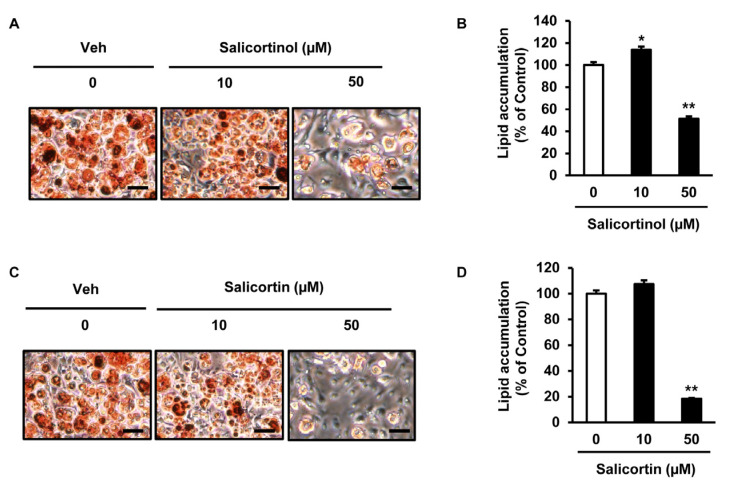
Inhibitory effect of salicortinol and salicortin on lipid accumulation in 3T3-L1 cells: (**A**) microscopy images of cells treated with different concentrations of salicortinol (as indicated) during adipocyte differentiation, followed by oil red O staining; (**B**) Quantification of intracellular lipid accumulation in salicortinol-treated 3T3-L1 cells; (**C**) microscopy images of cells treated with different concentrations of salicortin (as indicated) during adipocyte differentiation, followed by oil red O staining; and (**D**) Quantification of intracellular lipid accumulation quantification in salicortin-treated 3T3-L1 cells. B and D, *n* = 3 per group. The values represent the mean ± SEM. Scale bar = 100 µm. * *p* < 0.05, ** *p* < 0.01. Veh, vehicle (negative control).

To investigate whether salicortinol or salicortin had any effect on adipocyte differentiation, we analyzed lipid accumulation in 3T3-L1 cells in the presence of these molecules. Isolated compounds were dissolved in dimethyl sulfoxide, which was also used for the vehicle treatment (Veh; the negative control). Treatments with either salicortinol (10 or 50 µM) or salicortin (10 or 50 µM) were applied to cells for seven days during adipocyte differentiation. Cells were then stained for lipid content determination. Results showed that the number of Oil Red O-stained cells was significantly reduced by 50 µM salicortinol treatment (Figure 3A), showing a 48% decreased in lipid accumulation compared with that in Veh-treated cells (Figure 3B). In contrast, 10 µM salicortinol treatment slightly increased lipid accumulation compared with that in Veh treated cells (Figure 3A,B). Upon treatment with 50 µM salicortin, the number of cells stained with Oil Red O was remarkably reduced compared to that of cells treated with Veh (Figure 3C). In addition, lipid accumulation was not significantly affected by 10 µM salicortin, whereas it was decreased by 82% by 50 µM salicortin compared with that in cells treated with Veh (Figure 3D). Accordingly, we further studied the effect of salicortin on lipid accumulation in cells during adipogenesis as it showed a greater inhibitory effect than salicortinol did.

### 2.3. Salicortin Suppresses Adipogenesis by Attenuating the Expression of Lipogenic and Adipogenic Transcription Factors

**Figure 4:**
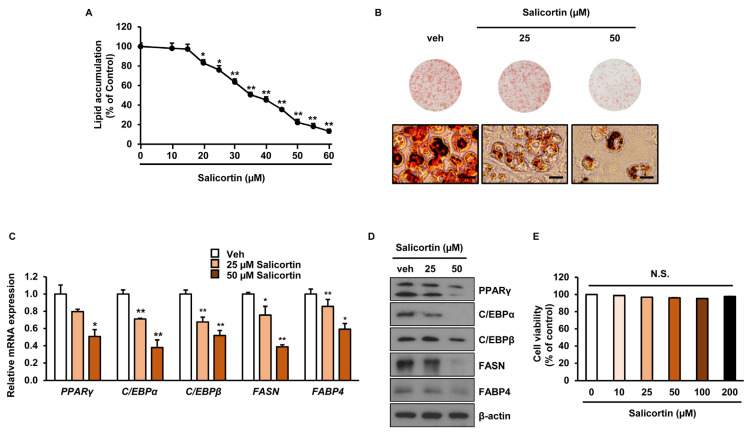
Inhibitory effect of salicortin on adipogenesis: (**A**) quantification of intracellular lipid accumulation in salicortin-treated 3T3-L1 cells compared to that in control cells; (**B**) Oil red O staining images (**top**) and microscopy (**bottom**) images in 3T3-L1 cells after 7 days of differentiation in either the absence or presence of salicortin at different concentrations. Scale bar = 100 µm; (**C**) gene expression quantification of PPARγ, FASN, FABP4, C/EBPβ, and C/EBPα, performed by real time qPCR; (**D**) immunoblot images of PPARγ, FASN, FABP4, C/EBPβ, and C/EBPα; and (**E**) evaluation of salicortin cytotoxicity. (**B**,**C**,**E**), *n* = 3 per group. N.S., not significant; Veh, vehicle (negative control). The values represent the mean ± SEM. * p < 0.05, ** *p* < 0.01.

The inhibitory effect of salicortin on lipid accumulation showed in a dose-dependent manner and the half inhibitory concentration (IC_50_) of salicortin was 37.1 µM (Figure 4A). We further assessed the effect of two concentrations (25 and 50 µM) of salicortin on adipocyte differentiation in detail. Oil Red O staining revealed that lipid droplets decreased in cells treated with 25 or 50 µM salicortin compared to those in the Veh treatment (Figure 4B). Furthermore, mRNA expression of adipogenic factors, including *proliferator-activated receptor gamma* (PPARγ), *CCAAT/enhancer binding protein* *β* (C/EBPβ)*, CCAAT/enhancer binding protein* *α* (C/EBPα), *lipogenic factor fatty acid synthase* (FASN), and *fatty acid binding protein 4* (FABP4) genes, was significantly reduced in cells treated with either 25 or 50 µM salicortin (Figure 4C). In addition, the protein levels of FASN, C/EBPα, PPARγ, C/EBPβ, and FABP4 were lower in cells treated with 25 and 50 µM salicortin that in control cells (Figure 4D). To determine whether the inhibitory effect of salicortin on adipogenesis involved cytotoxicity, we used the MTT assay. Cell viability was not affected by treatment with up to 200 µM salicortin (Figure 4E). These results suggest that salicortin suppresses lipid accumulation by reducing the mRNA and protein levels of lipogenic enzymes and adipogenic transcription factors in preadipocytes.

## 3. Discussion

Research on natural products extracted from medicinal plants has been actively carried out in recent years. Among compounds already isolated, some can inhibit adipocyte differentiation, showing their potential as anti-obesity agents [23,24]. However, the effect of salicortin isolated from *S. pseudolasiogyne* twigs on adipocyte differentiation has not yet been fully investigated. Here, we showed that salicortin suppresses adipocyte differentiation in 3T3-L1 cells.

Salicortin, a bioactive phytochemical identified in *S. pseudolasiogyne* twigs, is a salicin derivative with a 1-hydroxy-6-oxo-2-cyclohexenecarboxylate moiety, and it is dominant among secondary metabolites in a variety of medicinal plants, including *Populus* and *Salix* species. To date, several analogues of salicortin with acetyl groups in the sugar unit have been isolated and structurally identified, including 6′-*O*-acetylsalicortin, 2′-*O*-acetylsalicortin, 2′,6′-*O*-acetylsalicortin, and 3′-*O*-acetylsalicortin [13].

Adipogenesis is an important process that may lead to obesity [25]. The process though which adipocyte precursor cells differentiate and proliferate into mature adipocytes is called adipogenesis [26]. The differentiation of preadipocytes into mature adipocytes requires the expression of several transcription factors, including PPARγ, C/EBPβ, and C/EBPα [26]. These transcription factors play a major role in the expression of lipogenesis-related genes, such as FABP4 and FASN [27]. In this study, we found that salicortin, at concentrations of 25 and 50 µM, significantly inhibited lipid accumulation and decreased both the mRNA and protein levels of PPARγ, FASN, FABP4, C/EBPβ, and C/EBPα. Consistent with our results, 2′,6′-*O*-acetylsalicortin, identified as a salicortin analogue isolated from *S. pseudolasiogyne* twigs, also showed an anti-adipogenic effect through the suppression of the SREBP1c- and C/EBPα-dependent pathway [14]. In addition, salicortin-abundant *Populous balsamifera* extract exhibited an inhibitory effect on adipocyte differentiation in 3T3-L1 cells [28]. Although previous reports have shown the anti-adipogenic effect of salicortin isolated from *S. pseudolasiogyne* twigs [14], they only measured the IC_50_ ratio and did not reveal the molecular mechanism that underlies. In contrast, our results suggest that salicortin 25 µM not only suppressed lipid accumulation, but also downregulated the mRNA and protein levels of major adipogenic and lipogenic enzymes. Together, these findings suggest that salicortin derivatives can potentially inhibit adipocyte differentiation.

We showed that salicortin, with a 1-hydroxy-6-oxo-2-cyclohexenecarboxylate moiety, had a stronger inhibitory effect on lipid accumulation than salicortinol, which has a 1,6-dihydroxy-2-cyclohexenecarboxylate moiety. This finding suggests that the moiety in salicortin may determine the role of the molecule in adipogenesis. Consistent with our results, idescarpin isolated from the methanol extracts of *Idesia polycarpa* fruits has the 1-hydroxy-6-oxo-2-cyclohexenecarboxylate moiety and it showed the potent inhibitory activity on adipocyte differentiation with an IC_50_ value of 23.2 µM [29]. Furthermore, salicortin derivatives bearing the 1-hydroxy-6-oxo-2-cyclohexenecarboxylate moiety have exhibited a suppressive effect on adipocyte differentiation partially via inhibition of a C/EBPα dependent pathway [14]. Recently, salicortin has been reported to inhibit inducible nitric oxide synthase (iNOS) expression and nitric oxide (NO) production in LPS-stimulated RAW 264.7 cells [30]. Salicortin was also found to suppress TNF-α-induced ICAM-1 expression in human endothelial cells [31]. Moreover, salicortin was reported to suppress the LPS-induced inflammatory response through blockade of NF-κB and JNK MAPK signaling cascades in RAW 264.7 cells (a macrophage cell line) [32]. Though obesity is a chronic inflammatory state [33] and salicortin has anti-inflammatory properties [32], it is unclear whether salicortin has any potential efficacy on obesity-related metabolic deregulation. Our results show that salicortin directly inhibits adipogenesis, in addition to previously reported effects.

**Figure 5:**
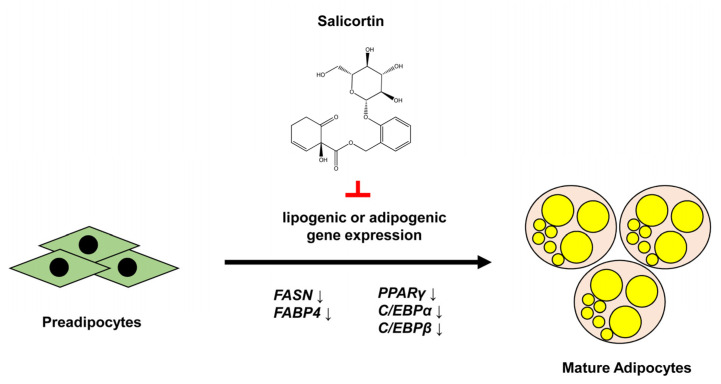
Model of mechanism of salicortin’s action. Salicortin suppresses adipocyte differentiation by reducing the mRNA and protein levels of lipogenic and adipogenic factors.

Collectively, our results demonstrate that salicortin isolated from *S. pseudolasiogyne* twigs inhibited adipocyte differentiation by reducing the protein and mRNA levels of adipogenic and lipogenic factors (Figure 5). Thus, it is necessary to further investigate the mechanism of action of salicortin as a novel natural anti-obesity compound.

## 4. Materials and Methods

### 4.1. General Experimental Procedures

Optical rotation was measured using a Jasco P-2000 polarimeter (Jasco, Easton, MD, USA). Ultraviolet (UV) spectra were acquired on an Agilent 8453 UV-visible spectrophotometer (Agilent Technologies, Santa Clara, CA, USA). NMR spectra were obtained using a Bruker AVANCE III 850 NMR spectrometer operating at 850 MHz (^1^H) and 212.5 MHz (^13^C; Bruker, Billerica, MA, USA). Preparative HPLC was performed using a Waters 1525 binary HPLC pump with a Waters 996 photodiode array detector (Waters Corporation, Milford, CT, USA) and an Agilent Eclipse C18 column (250 × 21.2 mm, 5 μm; flow rate: 5 mL/min; Agilent Technologies, Santa Clara, CA, USA). Semi-preparative HPLC was performed using a Shimadzu Prominence HPLC System with SPD-20A/20AV Series Prominence HPLC UV-Vis detectors (Shimadzu, Tokyo, Japan) and a Phenomenex Luna phenyl-hexyl column (250 × 10 mm inner diameter (ID), flow rate: 2 mL/min; Phenomenex, Torrance, CA, USA). LC/MS analysis was performed using an Agilent 1200 Series HPLC system equipped with a diode array detector and 6130 Series ESI mass spectrometer using an analytical Kinetex C18 100 Å column (100 × 2.1 mm, 5 μm; flow rate: 0.3 mL/min; Phenomenex, Torrance, CA, USA). Thin-layer chromatography (TLC) was conducted using precoated silica gel F_254_ plates and RP-18 F_254s_ plates (Merck). TLC spots were detected using UV light and heating after dipping in anisaldehyde–sulfuric acid.

### 4.2. Plant Material

*S. pseudolasiogyne* twigs were collected in Chungcheongnam-do, Republic of Korea in June 2021. A voucher specimen (HIMH-2109) was identified by Dr. Hye-Ryen Na at the Northeastern Asia Biodiversity Institute, Seoul 05677, Republic of Korea. The material was deposited in the herbarium of Nakdonggang National Institute of Biological Resources, Sangju, Republic of Korea.

### 4.3. Plant Extraction and Isolation of Compounds

*S. pseudolasiogyne* twigs (1.6 kg) were dried at 35–45 °C in a plant drying oven for one week, pulverized, and sonicated three times with 80% ethanol (10 L) for 90 min each at room temperature. The resulting ethanol extract was evaporated in vacuo to obtain a crude brown ethanol extract (123.6 g). An extract sample (9.1 g) was dissolved in distilled water (700 mL) for solvent partitioning using CH_2_Cl_2_ to remove wax, lipids, and fatty acids, and the residue was concentrated using an evaporator to yield the crude extract (4.0 g). The crude extract (1.0 g) was separated using preparative reversed-phase HPLC (from 30% MeOH to 50% MeOH for 80 min, gradient system) to obtain four fractions (P1–P4). Fraction P4 (306 mg) was isolated by semi-preparative reversed-phase HPLC using 39% MeOH with a Phenomenex Luna C18 column to yield compounds **1** (0.7 mg, t_R_ = 33.0 min, ESIMS (positive-ion mode) *m*/*z* 427 [M + H]^+^) and **2** (1.7 mg, t_R_ = 37.5 min, ESIMS (positive-ion mode) *m*/*z* 425 [M + H]^+^).

### 4.4. Compounds Treatment

Isolated compounds salicortinol and salicortin were dissolved in dimethyl sulfoxide (DMSO). The stock concentration of salicortinol and salicortin was 10 mM. Salicortinol was used at concentrations of 10 and 50 µM. Salicortin was used at concentrations of 10, 25, and 50 µM. DMSO was used as a vehicle (Veh) for treatment. When the cells were treated with the compounds, the percentage values of DMSO present in the final solution of compounds exposed to the cells were below 0.5%.

### 4.5. Adipocyte Differentiation and Maintenance

Dulbecco’s modified Eagle medium (DMEM) supplemented with 10% bovine serum (cat. 26170043), and 1% penicillin-streptomycin was used to maintain 3T3-L1 preadipocytes. For triggering differentiation, the medium was changed for differentiation medium (DMI) containing DMEM, 10% fetal bovine serum (FBS), 1 µM dexamethasone, 0.5 mM IBMX, and 5.0 µg/mL insulin. DMI was substituted by DMEM with 10% FBS and insulin after two days and then replaced every 2 days until cells were fully differentiated (after 7 days) [34].

### 4.6. Oil Red O Staining

Oil red O staining was performed on fully differentiated adipocytes. Before staining, cells were washed with phosphate-buffered saline (PBS) followed by formalin (10%). The cells were then incubated with new formalin (10%) for at least 1 h. After discarding the incubation solution, isopropanol (60%) was added for 5 min and cells were completely dried. The cells were then treated with oil red O solution (Sigma, St. Louis, MO, USA, cat. O0625) for 10 min. and washed five times with distilled water to clear the background. After staining, the cells were observed under a microscope. To quantify lipid droplets, isopropanol (100%) was added to stained cells for at least 1 h and measured with a spectrophotometer at 500 nm [35].

### 4.7. Extraction of Proteins and Western Blotting

3T3-L1 cells were washed with PBS and lysed using radioimmunoprecipitation assay buffer containing a protease inhibitor cocktail (Roche, Basel, Switzerland, cat. 11697498001), 0.1 M NaF, 2 mM Na_3_VO_4_, and 0.25% deoxycholate. The lysate was left 30 min on ice and centrifugated for protein collection. Protein concentrations were calculated using the Bio-Rad protein assay dye reagent (Bio-Rad, Hercules, CA, USA, cat. 5000006). Protein separation was assessed by SDS-PAGE and transferred to polyvinylidene difluoride membranes. Bovine serum albumin (5%) was used to block the membranes. Primary antibodies were maintained overnight in a cold room using a shaking machine. Primary antibodies against C/EBPα, C/EBPβ, FASN, FABP4, and PPARγ were purchased from Cell Signaling Technology. After incubation with the primary antibodies, the cells were washed three times for 10 min each with Tris-buffered saline containing Tween 20, followed by incubation with goat anti-rabbit horseradish peroxidase-conjugated IgG for 1 h. The ECL western blotting system (Thermo Fisher Scientific, Waltham, MA, USA, cat. 34580) was used to develop the expected protein signals [36].

### 4.8. Isolation of RNA and Quantitative Polymerase Chain Reaction (qPCR)

Total RNA was isolated using TRIzol reagent (Thermo Fisher Scientific, cat. 15596026). RNA, 1 μg, was utilized for the synthesis of complementary DNA (cDNA) using random hexamer primers and SuperScript II reverse transcriptase. Quantitative polymerase chain reaction (qPCR) was then performed using newly synthesized cDNA as template [37]. The primers used are listed in Table 1.

### 4.9. Statistical Analysis

Data are presented as the mean ± standard error of the mean (SEM). Statistical analyses were performed using Student’s *t*-test, and *p*-value < 0.05 was considered statistically significant.

## 5. Conclusions

In this study, the investigation of the composition of the EtOH extract of *S. pseudolasiogyne* twigs led to the isolation of two salicin derivatives, salicortinol and salicortin. Both salicortinol and salicortin inhibited lipid accumulation in 3T3-L1 cells. Interestingly, salicortin had a stronger inhibitory effect on adipocyte differentiation, and provoked a reduction in the mRNA and protein levels of several adipogenic and lipogenic transcription factors. These findings suggest that salicortin may be a potential compound for the prevention or treatment of obesity by inhibiting adipocyte differentiation.

## Figures and Tables

**Table 1:** Primers used for real-time qPCR.

Name	Forward (5′→3′)	Reverse (5′→3′)
PPARγ	GGGTGAAACTCTGGGAGATTCTCC	CAGCAACCATTGGGTCAGCTCT
C/EBPα	ACAACATCGCGGTGCGCAAGA	TGCCATGGCCTTGACCAAGGAG
C/EBPβ	GTCCAAACCAACCGCACAT	CAGAGGGAGAAGCAGAGAGTT
FASN	CGGAAACTGCAGGAGCTGTC	CACGGAGTTGAGCCGCAT
FABP4	TGGAAGCTTGTCTCCAGTGA	AATCCCCATTTACGCTGATG
GAPDH	GTCTTCCTGGGCAAGCAGTA	CTGGACAGAAACCCCACTTC

## Data Availability

Not applicable.

## Supplementary Materials

The following materials are available online at https://www.mdpi.com/article/10.3390/molecules27206954/s1: Figure S1: ^1^H NMR spectrum of **1** (CD_3_OD, 850 MHz); Figure S2: ^1^H NMR spectrum of **2** (CD_3_OD, 850 MHz).
