# Supplementary material for: Anti-Adipogenic Effects of Salicortin from the Twigs of Weeping Willow (Salix pseudolasiogyne) in 3T3-L1 Cells
Source: Molecules. 2022 Oct 17;27(20):6954. doi: 10.3390/molecules27206954 (PMC9609119; doi:10.3390/molecules27206954)

## Supplementary data

# Anti-adipogenic Effects of Salicortin from *Salix pseudolasiogyne* Twigs in 3T3-L1 Cells

Hee Jung Kim [1,2,†], Da Eun Lee [3,†], Eon Chung Park [3], Moon-Jin Ra [4], Sang-Mi Jung [4], Jeong-Nam Yu [5], Sung Hee Um [1,2,6,*], and Ki Hyun Kim [3,*]

[1] Department of Molecular Cell Biology, Samsung Biomedical Research Institute, Sungkyunkwan University School of Medicine, Suwon, Gyeonggi-do, 16419, Republic of Korea
[2] Department of Health Sciences and Technology, Samsung Advanced Institute for Health Sciences and Technology, Samsung Medical Center, Sungkyunkwan University, Seoul, 06351, Republic of Korea
[3] School of Pharmacy, Sungkyunkwan University, Suwon 16419, Republic of Korea
[4] Hongcheon Institute of Medicinal Herb, Hongcheon-gun, Gangwon-do 25142, Republic of Korea
[5] Nakdonggang National Institute of Biological Resources, Sangju, Gyeongsangbuk-do 37242, Republic of Korea
[6] Biomedical Institute Convergence at Sungkyunkwan University, Suwon, Gyeonggi-do, 16419, Republic of Korea
* Correspondence: shum@skku.edu (S.H.U.); khkim83@skku.edu (K.H.K.); Tel.: +82-31-299-6123 (S.H.U.); +82-31-290-7700 (K.H.K.)
† These authors contributed equally to this study

**Figure S1.** $^1$H NMR spectrum of **1** ($CD_3OD$, 850 MHz)

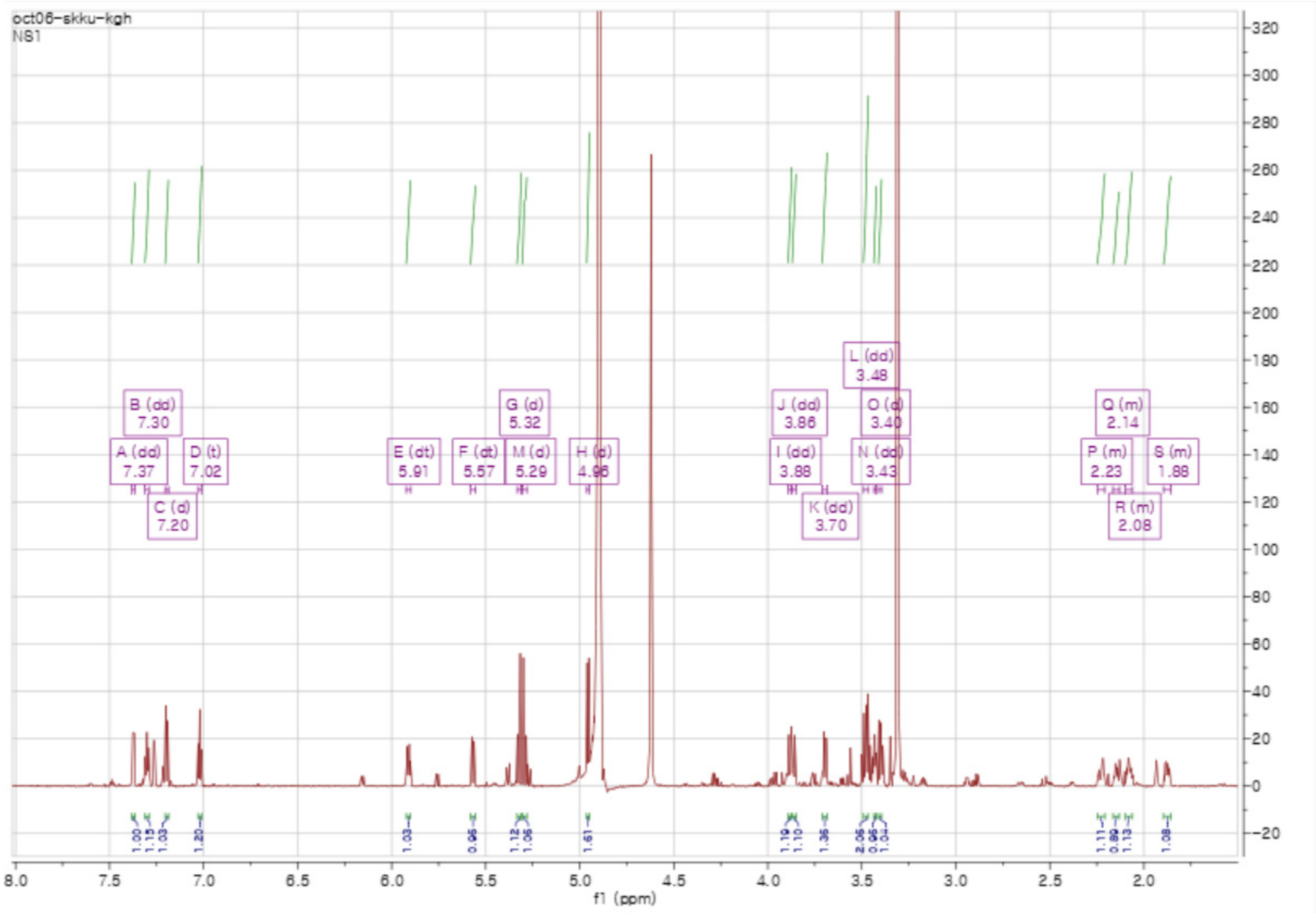

**Figure S2.** $^{1}$H NMR spectrum of **2** ($CD_3OD$, 850 MHz)

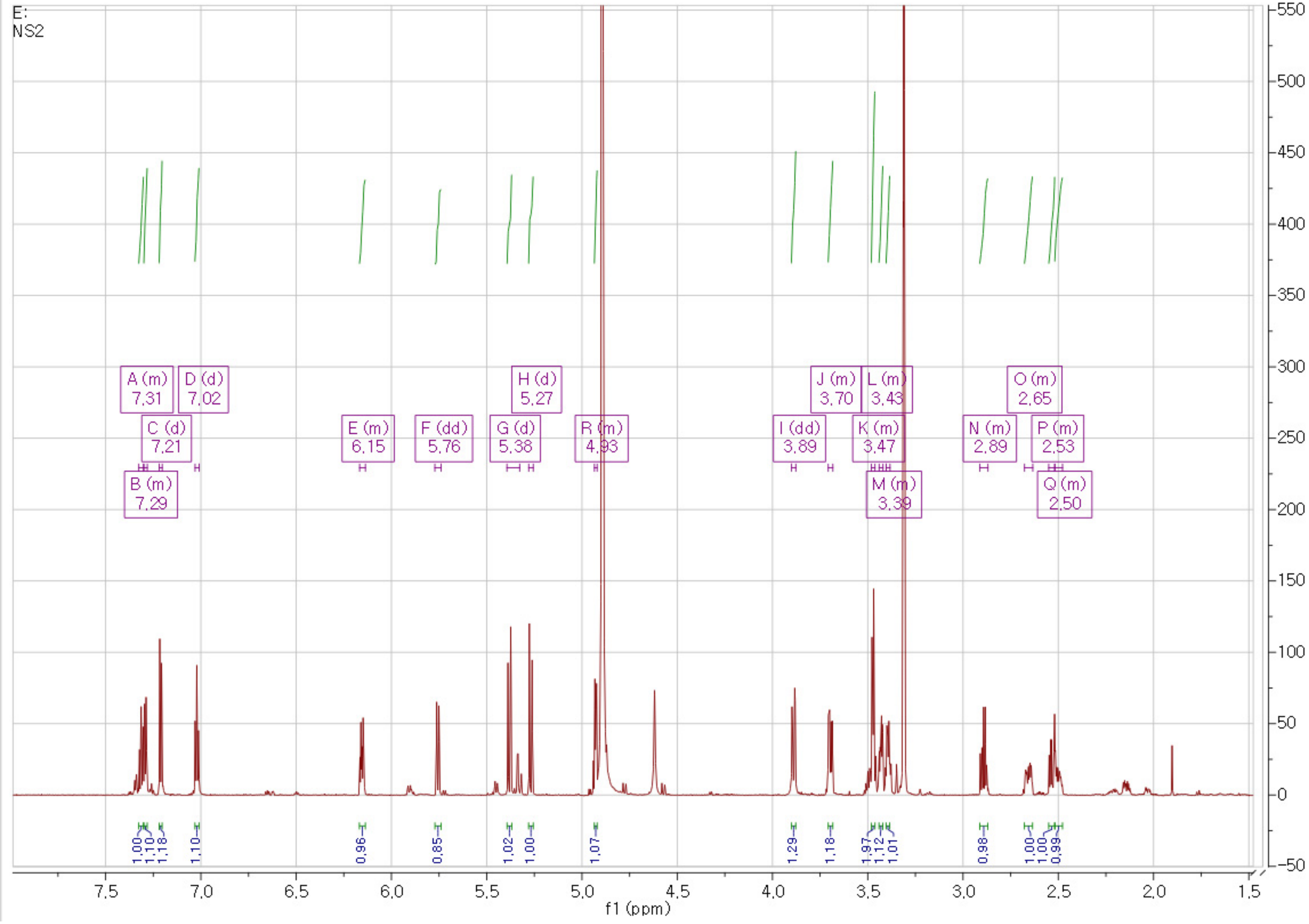

Supplement: Supplementary file 1 [file molecules-27-06954-s001.zip › molecules-1901582-supplementary.pdf]
